# Supplementary material for: Graph transformer for ancient ancestry inference
Source: bioRxiv. 2026 Apr 7:2026.04.05.714076. Preprint. [Version 1] doi: 10.64898/2026.04.05.714076 (PMC13082007; doi:10.64898/2026.04.05.714076)
Supplement: Supplement 1 [file media-1.pdf]

Supplementary Figures

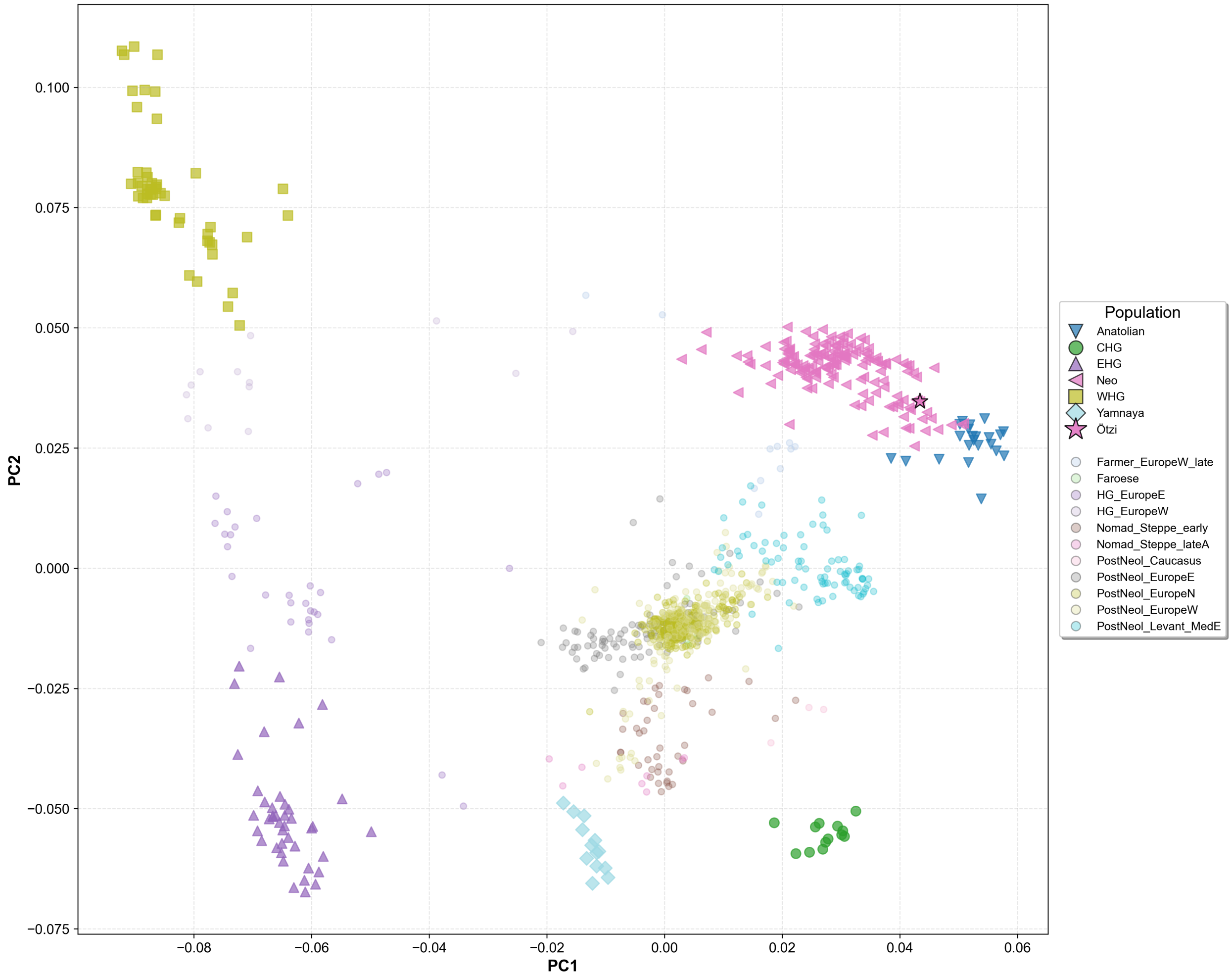

Supplementary Figure S1: PCA of ancient DNA samples. Indicated in bold shapes are references used for local ancestry with ARGMix.

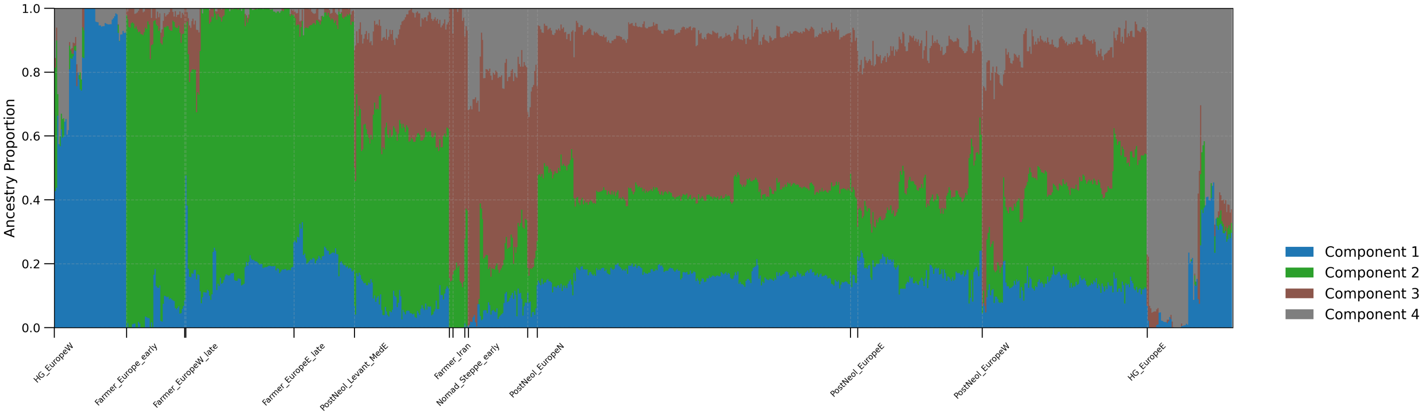

Supplementary Figure S2: Plot of ADMIXTURE unsupervised clustering run on ancient DNA samples with K=4.

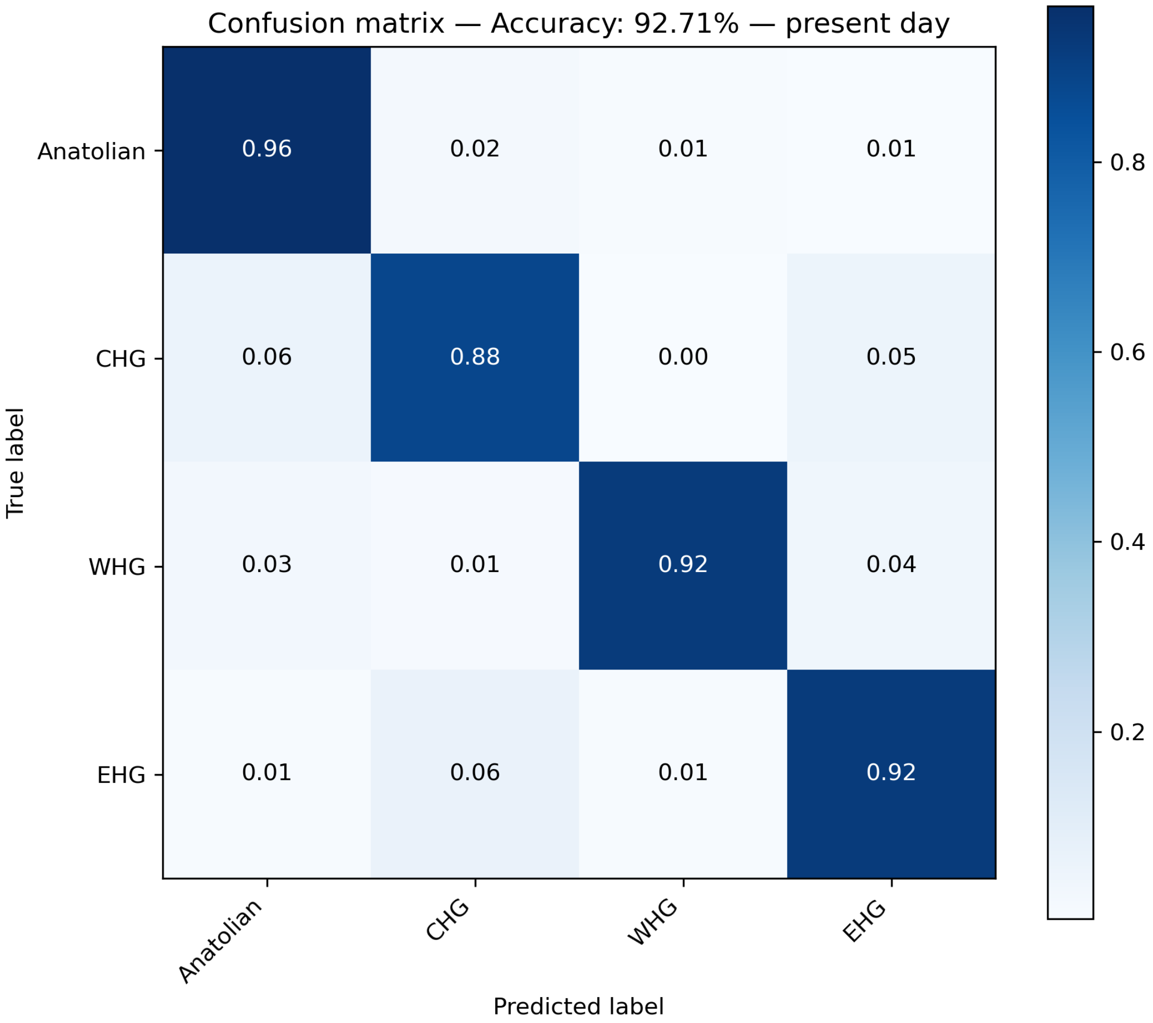

Supplementary Figure S3: Confusion matrix of present-day Europeans for worst-performing misspecified demography nevertheless showing robust accuracy.

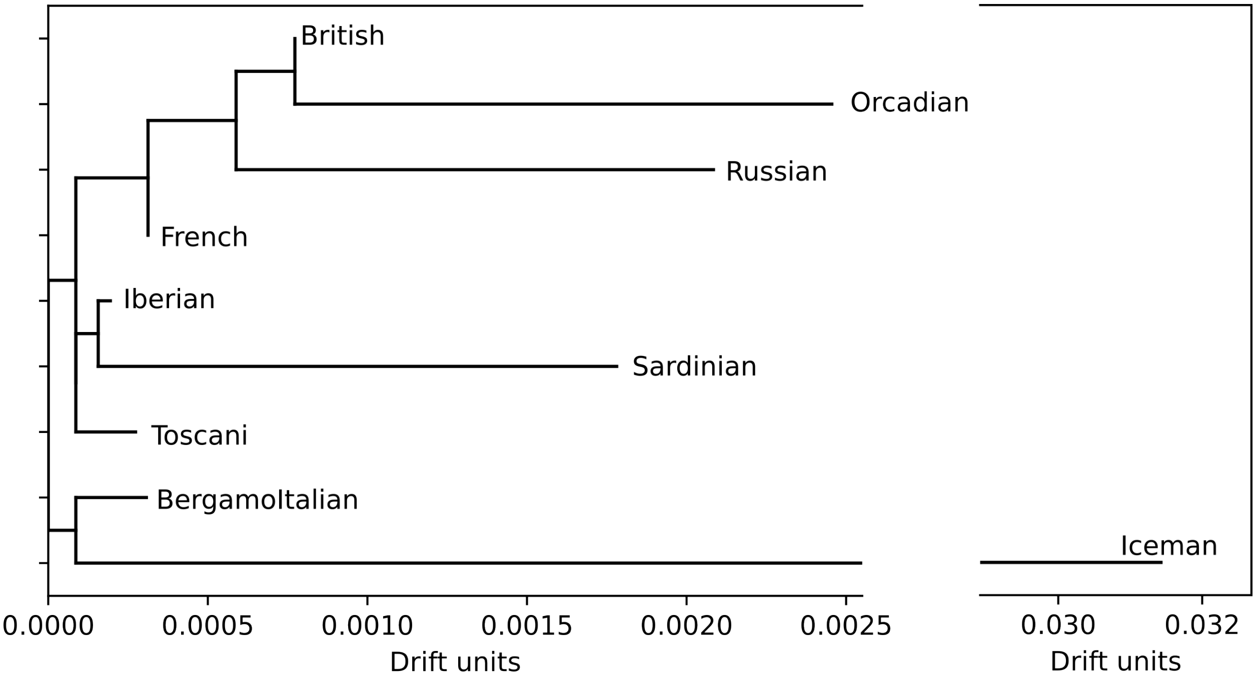

Supplementary Figure S4: Anatolian-specific Treemix results with zero migration edges shows Ötzi the Iceman sharing a clade with modern day northern Italians (Bergamo Italians).

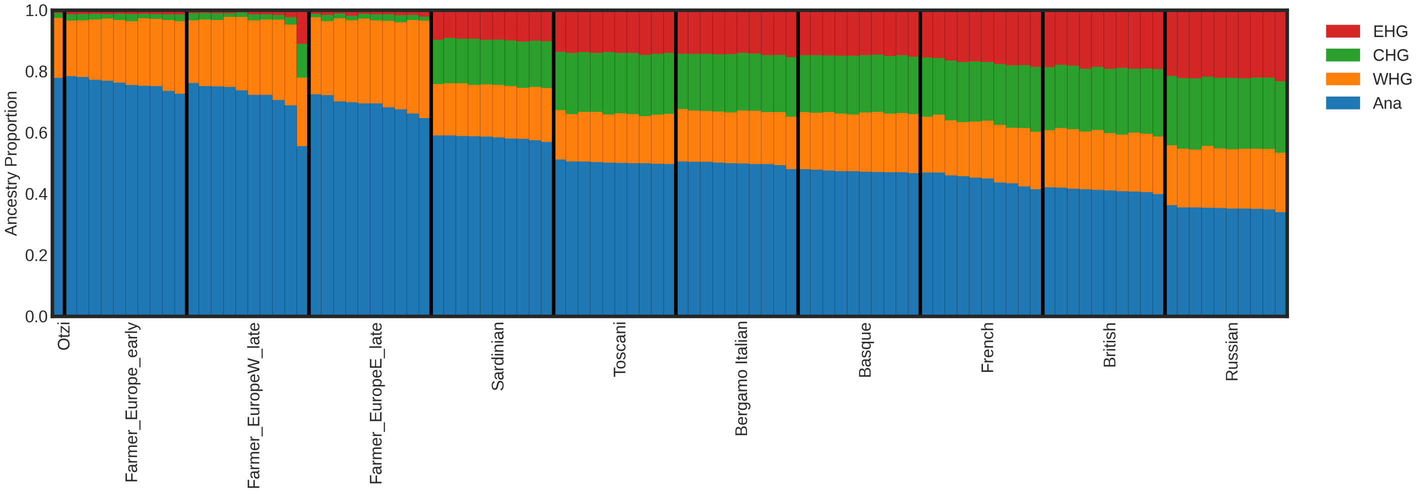

Supplementary Figure S5: Admixture proportions determined by summing of local ancestry calls from ARGMix. Present-day Europeans are included along with early and late Neolithic farmers. Populations are sorted by average Anatolian proportion.

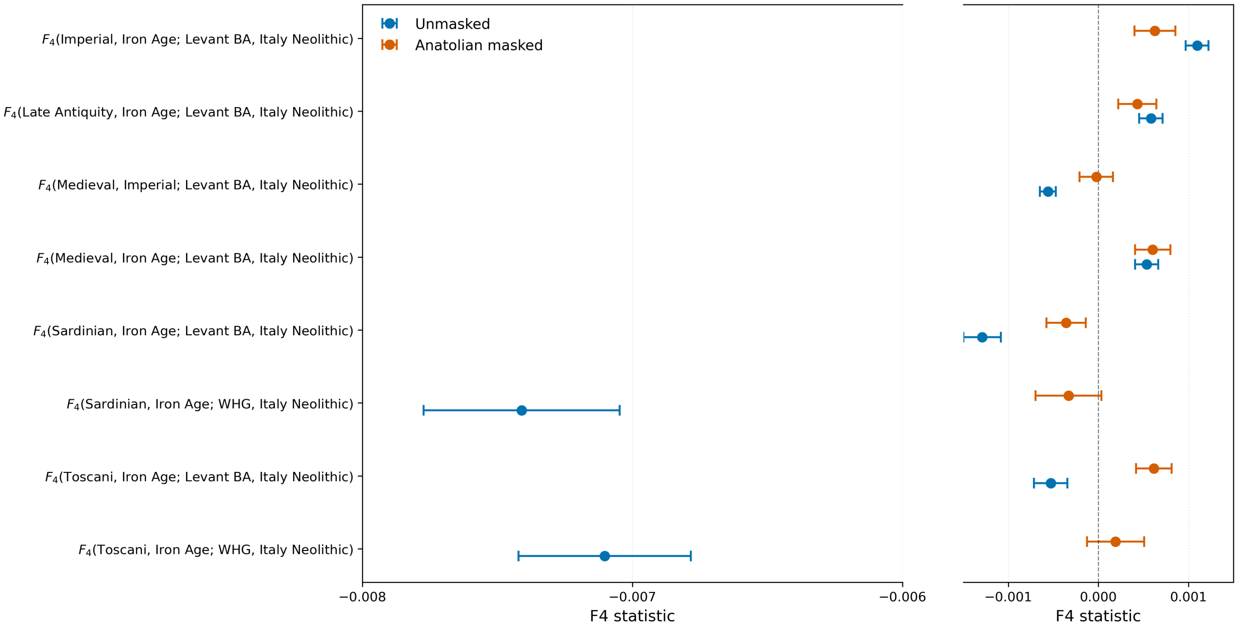

Supplementary Figure S6: Changes in F4 statistics after masking for Anatolian ancestry. Imperial and Iron Age refer to Imperial Italians and Iron Age Italians.
